# Development of a scalar-based geometric parameterization approach for the crystal structure landscape of dithienylethene-based crystalline solids

**DOI:** 10.1107/S2052252523008060

**Published:** 2023-09-26

**Authors:** Travis B. Mitchell, Xiaotong Zhang, Ronald T. Jerozal, Yu-Sheng Chen, SuYin Wang, Jason B. Benedict

**Affiliations:** a Department of Chemistry, University at Buffalo, State University of New York, Buffalo, NY 14260-3000, USA; b NSF’s ChemMatCARS, University of Chicago, Chicago, Lemont, IL 60439, USA; Sun Yat-Sen University, China

**Keywords:** di­aryl­ethene, photoactive, crystal landscapes, crystal engineering, properties of solids, crystal design

## Abstract

Dithienylethenes (DTEs) are a promising class of organic photoswitches that can be used to create crystalline solids with properties controlled by light. However, the ability of DTEs to adopt multiple conformations, only one of which is photoactive, complicates the rational design of these materials. Herein, the synthesis and structural characterization of 19 crystalline solids containing a single DTE molecule are described. A novel *D*–*D* analysis of the molecular geometries obtained from rotational potential energy surface calculations and the ensemble of experimental structures were used to construct a crystal landscape for DTE. Of the 19 crystal structures, 17 contained photoinactive DTE rotamers and only 2 were photoactive. These results highlight the challenges associated with the design of these materials. Overall, the *D*–*D* analysis described herein provides rapid, effective and intuitive means of linking the molecular structure to photoactivity that could be applied more broadly to afford a general strategy for producing photoactive di­aryl­ethene-based crystalline solids.

## Abstract 2

The crystal structure landscape of (*Z*)-1,2-bis­[2-methyl-5-(pyridin-4-yl)thio­phen-3-yl]-1,2-di­phenyl­ethene (DTE) was determined using a combination of computational and experimental molecular geometries. A novel *D*–*D* analysis was developed which provides a rapid, effective and intuitive means of visualizing the crystal landscape and assessing the conformer type present in and photoactivity of the resulting crystalline solids.

## 1. Introduction

Crystal engineering is a powerful tool used to design and synthesize new materials with specific physical and chemical properties by exploiting the role that individual intermolecular interactions play when forming crystalline solids (Nangia & Desiraju, 2019 ▸). Without a doubt, crystal engineering has been central to the development of crystalline materials tailored for a wide range of chemical applications including pharmaceuticals (Karimi-Jafari *et al.*, 2018 ▸; Duggirala *et al.*, 2016 ▸; Guo *et al.*, 2021 ▸), biosensors (Fu *et al.*, 2023 ▸), gas separation and storage (Shekhah *et al.*, 2018 ▸; Cheng *et al.*, 2022 ▸), and electronics and optics (Kandambeth *et al.*, 2022 ▸; Jiang *et al.*, 2021 ▸; Dar & Rashid, 2021 ▸).

Traditionally, crystal structures are viewed as repeating patterns of atoms, molecules or ions arranged in a three-dimensional lattice. However, a particularly powerful concept in crystal engineering is to instead view the same crystal structure as a rich structural landscape in which the position of a molecule in a single crystal is a data point on this landscape (Desiraju, 2021 ▸). Different crystallizing conditions can lead to the formation of different crystal structures, each with its own unique set of properties. Therefore, it is important to assess a wide variety of crystallizing conditions to produce crystalline solids (Samanta *et al.*, 2021 ▸; Tothadi & Desiraju, 2012 ▸; Singh *et al.*, 2016 ▸; Ranjan *et al.*, 2020 ▸; Rajkumar & Desiraju, 2021 ▸). Consequently, a wide variety of crystalline solids including polymorphs, hydrates, solvates, co-crystals and metal–organic frameworks can help deepen our understanding of how the molecule of interest is impacted by the surrounding crystalline environment and vice versa. This includes identifying supramolecular synthons and non-covalent interactions that are important in the formation of crystals, which inevitably affects the physical and chemical properties of crystalline solids, such as their solubility and melting point.

The changing crystalline environments directly impact the molecule of interest and ultimately the functionally important properties of the crystal, including water sorption (Reutzel-Edens & Bhardwaj, 2020 ▸), electronic properties (Narayanan *et al.*, 2017 ▸; Sun *et al.*, 2019 ▸), detonation properties (Landenberger *et al.*, 2015 ▸; Kent *et al.*, 2018 ▸; Aakeröy *et al.*, 2015 ▸ *b*) and thermal stability (Aakeröy *et al.*, 2015*a* ▸; Angevine *et al.*, 2022*b* ▸,*a* ▸) among others. Likewise, photoactivity in the solid state is another property heavily influenced by crystal structure (Yelgaonkar *et al.*, 2020 ▸; Campillo-Alvarado *et al.*, 2020 ▸; Borchers *et al.*, 2022 ▸).

Dithienylethenes (DTEs) are a promising class of molecules for use in organic solid-state photoswitches. DTEs are attractive because they are solid-state reactive, both isomers are thermally stable and they are fatigue resistant (Fukaminato *et al.*, 2001 ▸; Herder *et al.*, 2015 ▸; Kitagawa *et al.*, 2013 ▸; Kobatake *et al.*, 2000 ▸, 2002 ▸, 2007 ▸; Shibata *et al.*, 2002 ▸). However, the rational design process for DTE-based crystalline solids is complicated by their conformational flexibility. The two thio­phene rings can rotate around the central ethyl­ene bridge, which gives rise to parallel and anti-parallel rotational isomers (Zhang & Tian, 2018 ▸; Rice *et al.*, 2020 ▸; Cox *et al.*, 2016 ▸). DTE molecules are considered to be potentially photoactive in the solid state when they adopt the antiparallel geometry and have an interatomic distance between the two active carbon atoms of less than 4.2 Å (*see infra*) (Kobatake *et al.*, 2002 ▸). If neither of these conditions are met, the molecule is not expected to be photoactive. That said, the geometry of the DTEs within the crystalline structure profoundly affects their functionality as a photoswitch. Thus, the development and assessment of the crystal structure landscape for these materials will play a critical role in the rational design of future DTE-based materials with desired molecular geometries.

**Figure 1:**
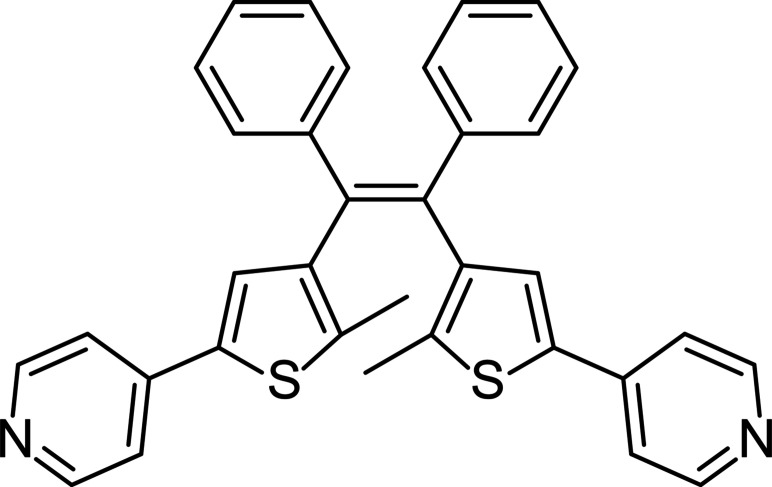
Chemical structure of (*Z*)-1,2-bis­(2-methyl-5-(pyridin-4-yl)thio­phen-3-yl)-1,2-di­phenyl­ethene (DTE).

Herein we report the synthesis of (*Z*)-1,2-bis­(2-methyl-5-(pyridin-4-yl)thio­phen-3-yl)-1,2-di­phenyl­ethene (DTE), a bulky acyclic DTE molecule with a pyridyl functionalized thio­phene pendant groups (Fig. 1 ▸). We incorporated DTE into 19 different crystalline solids such as polymorphs, co-crystals, coordination polymers and metal–organic frameworks. Single-crystal X-ray diffraction (SCXRD) analysis revealed that only 2 of the 19 crystal structures contained DTE in the photoactive geometry. These findings led to the development of a novel scalar-based approach to parametrizing the DTE geometry, which may be applied more broadly to this class of photoactive molecules.

## 2. Methods

### 2.1. Synthesis and characterization

All reagents were purchased from commercial sources and used as received unless otherwise noted. ^1^H NMR spectra were obtained on a Varian Inova-400 or Varian Inova-500 operating at 400 and 500 MHz, respectively. ^1^H NMR peaks are referenced to residual solvent signals in CDCl_3_ at 7.26 p.p.m. (Gottlieb *et al.*, 1997 ▸). SCXRD data were collected on a rotating anode source or using synchrotron radiation. Full details of the synthesis and characterization are provided in the supporting information (Fig. S1) which includes a detailed list of co-formers and linkers used for synthesizing crystals (Fig. S2).

### 2.2. Nomenclature

Each of the 19 crystal structures comprise one or two crystallographically unique DTEs. The following naming convention will be used to differentiate between these structures and their crystallographically unique DTEs. Each crystal structure is assigned a number *X* and labelled **DTE-[*X*]**. If **DTE-[*X*]** has one crystallographically unique DTE in the asymmetric unit, DTE itself will be referred to as **DTE-*X***. If **DTE-[*X*]** has two crystallographically unique DTEs in the asymmetric unit, **DTE-*X*a** or **DTE-*X*b** will be used to refer to individual DTEs, while **DTE-*X*a/b** will be used to refer to both DTEs.

### 2.3. Computational methods

All calculations were performed using density functional theory (DFT) with the computational software suite *Gaussian09* (Revision D.01; Frisch *et al.*, 2013 ▸). The hybrid functional ωB97X-D with the 6-31G(d) basis set was used for all calculations (Chai & Head-Gordon, 2008 ▸). DTE and the methyl-substituted variation were geometry optimized in the anti-active geometry. The vibrational frequencies of each optimized DTE were calculated to verify that each geometry was a true energetic minimum.

Relaxed potential energy surface (RPES) scans were performed on the optimized DTE and its methyl-substituted variant. The torsion angle Φ_A_[C18, C1, C8, C11] was rotated by ±1° increments creating a clockwise (CW, −1°, negative) or counter-clockwise (CCW, +1°, positive) rotation about the C1—C8 bond (Fig. 1 ▸). After each 1° increment, a geometry optimization was performed on the molecule while keeping Φ_A_ rigid. These rotation and geometry optimization processes were performed in succession until all values of Φ_A_ within ±180° were examined for a total of 360°. All resulting energies were converted from Hartree to kcal mol^−1^.

**Figure 2:**
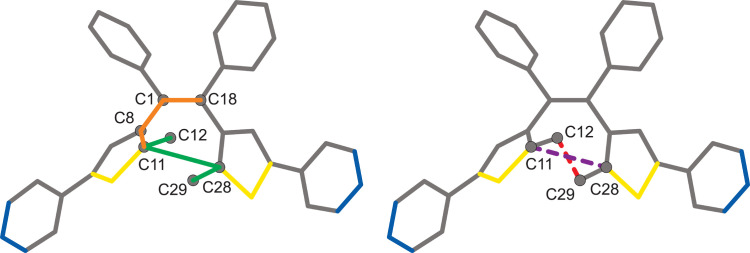
Representation of DTE identifying the atoms used to calculate the parameters φ_A_ (orange solid line), φ_B_ (green solid line), *D*_Me-Me_ (red dash line) and *D*_active_ (purple dash line).

In addition to Φ_A_, three other parameters were used to assess the geometries of DTE (Fig. 2 ▸): the relative orientation of methyl groups at the active carbon atoms (Φ_B_), the interatomic distance between the active carbon atoms (*D*_active_) and the interatomic distance between the methyl carbon atoms bonded to the active carbon atoms (*D*_Me-Me_). The relative orientation of methyl groups at the active carbon atoms is defined as the torsion angle Φ_B_[C12, C11, C28, C29]. *D*_active_ is defined as the interatomic distance between carbon atoms C11 and C28. *D*_Me-Me_ is defined as the interatomic distance between carbon atoms C12 and C29.

## 3. Results and discussion

### 3.1. Summary of crystal structures obtained

A total of 19 **DTE-[1–19]** single crystals containing DTE were analysed using SCXRD. Two neat crystal structures of DTE (**DTE-[1,2]**) are polymorphs. Four crystal structures (**DTE-[3–6]**) are co-crystals with multitopic carb­oxy­lic acids. Four crystal structures (**DTE-[7–10]**) are isostructural coordination polymers with the metals cobalt, nickel, zinc and cadmium. Crystal structures **DTE-[11,12]** are two unique coordination polymers with copper coordinated to iodine and bromine, respectively. The remaining crystal structures (**DTE-[13–19]**) were produced from MOF syntheses in which zinc and DTE were solvothermally reacted with various multitopic carb­oxy­lic acids. Five of the crystal structures (**DTE-[3,4,6,14,15]**) possess two crystallographically unique DTEs. Altogether, the present work includes a total of 24 unique DTE geometries arising from 19 crystal structures. By visual inspection, 6 of the molecules (**DTE-4a/b,5,6a/b,11)** were assigned to the P3/P4 conformation. A total of 16 molecules, **DTE-1,2,3a/b,7–10,12,13,14*a*/b,15*a*/b,16,17**, were assigned the AP2 conformation, and the two remaining molecules, **DTE-18,19**, were assigned the AP1 conformation. The conformer assignments and structural parameters for the experimental DTE geometries are provided in Table S1 of the supporting information. Additionally, the photoactivity of each crystal structure was confirmed by irradiating a single crystal with UV light. The single crystal was deemed photoactive if it showed any observable colour change, otherwise it was deemed photoinactive.

### 3.2. Relaxed potential energy scan

**Figure 3:**
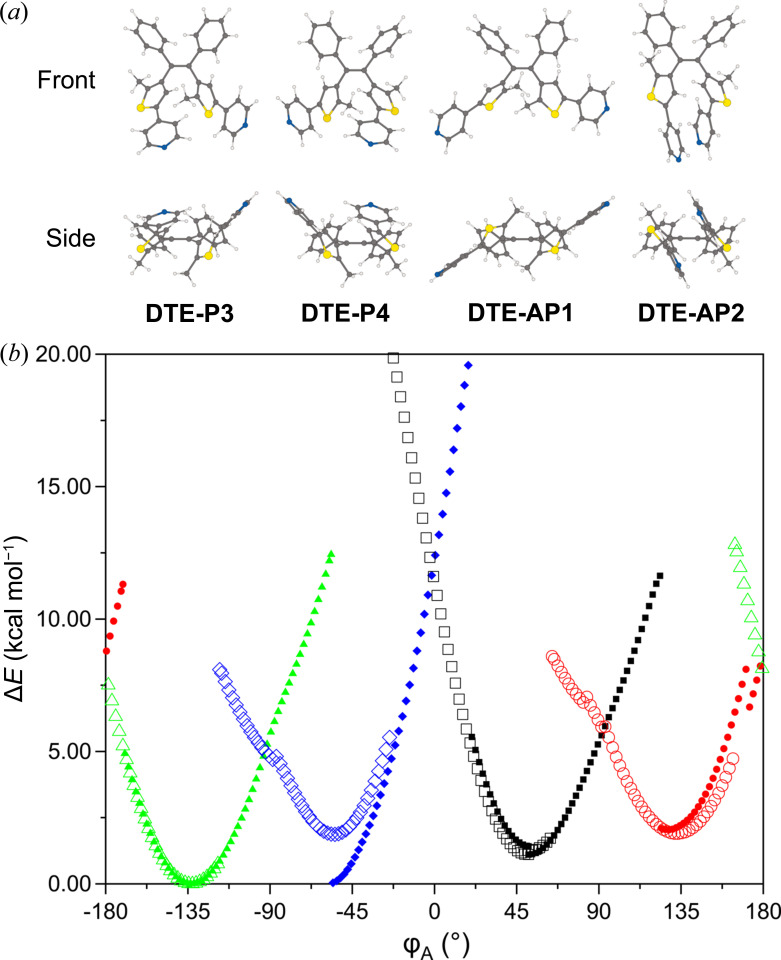
(*a*) The four (+)-DTE conformers viewed from front and side perspectives. (*b*) RPES plot for DTE. Filled shapes represent +1° increments, while empty shapes represent −1° increments. AP1 (black, square), AP2 (red, circle), P3 (green, triangle) and P4 (blue, diamond).

The RPES plot for **DTE** reveals eight energetic minima and eight energetic maxima split evenly between each scan direction [Fig. 3 ▸(*b*)]. The four sets of energetic minima correspond to four discrete conformers: two conformers with antiparallel geometry (**DTE-AP1** and **DTE-AP2**) and two conformers with parallel geometry (**DTE-P3 and DTE-P4**). Fig. 3 ▸(*a*) depicts each geometry from 2 different perspectives. The orientation of the vectors 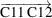 and 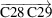 characterizes each geometry. The antiparallel geometry is characterized by the two vectors pointing in approximately opposite directions, whereas the parallel geometry occurs when two vectors point in a similar direction.

The torsion angles (Φ_A_) and energies relative to the lowest-energy conformer **DTE-P3** (Δ*E*) are summarized in Table 1 ▸. These four conformers will be used to analyse the conformers observed in the single-crystal structures.

The **DTE-AP1** and **DTE-AP2** conformers can be interconverted by rotating each thienyl moiety in the same direction. This concerted rotation of the thienyl moieties results in Φ_A_ increasing from 51.46° for **DTE-AP1** to 127.46° for **DTE-AP2**. Consequently, *D*_active_ also increases during this process from 3.45 Å for **DTE-AP1** to 5.19 Å for **DTE-AP2**. The photoactivity of dithienylethenes is well established and requires the antiparallel geometry and for *D*_active_ to be less than 4.2 Å. Though **DTE-AP1** and **DTE-AP2** have the correct geometry necessary to be photoactive, only **DTE-AP1** is potentially photoactive (*D*_active_ = 3.45 Å). This indicates that *D*_active_ is the most significant distinction in photoactivity between the two antiparallel conformers. The two parallel conformers can be interconverted in a similar way to the two antiparallel conformers. Although *D*_active_ is less than 4.2 Å for the two parallel conformers (**DTE-P3** and **DTE-P4**), their geometry prohibits any photoactivity.

The energy differences between each scan direction are 0.00 kcal mol^−1^ for **DTE-AP1**, 0.15 kcal mol^−1^ for **DTE-AP2**, 0.00 kcal mol^−1^ for **DTE-P3** and 1.83 kcal mol^−1^ for **DTE-P4**. The larger energy difference between each scan direction for **DTE-P4** is caused by a difference in the degree of planarity between the pyridyl rings and thio­phene rings (Fig. S3).

An RPES scan on a similar molecule in which the pyridyl rings were substituted with methyl groups resulted in no significant scan-dependent differences in energy for any of the conformers (Table S2, Fig. S4). Furthermore, the Δ*E* values for the two methyl-substituted parallel conformers (P3 and P4) were almost identical at ∼0.36 kcal mol^−1^. Thus, the **DTE-P3** and **DTE-P4** conformers will be generally described as a singular species of quasi-enantiomers and labelled **DTE-P3/P4**.

The increase in energy of each conformer as they approach their respective energetic maximum is caused by an increasingly unfavourable steric interaction between the phenyl rings on the backbone and the methyl group bonded to the active carbon. This interaction is seen as the phenyl rings and methyl groups rotating in a concerted effort to reduce their interaction with each other. Smaller energetic barriers are observed between the anti–anti and para–para rotamers, whereas the larger barriers are observed between the anti–para and para–anti geometries.

### 3.3. DTE scalar-based parameterization

The DTE conformer type is critically important to the photoactivity of a crystalline solid and has been traditionally assigned by visually inspecting the DTE molecule itself. However, this method can be time-consuming and subjective. To address this issue, we developed a series of DTE parameters (*D*_active_, *D*_Me-Me_ and Φ_B_) that can be quickly calculated and used to unambiguously determine the conformer type and visualize the crystal landscape.

The first parameter, *D*_active_, provides some information, but it is not enough information to unambiguously determine the type of conformer. As mentioned earlier, the photoactivity of DTE molecules depends on both the molecular geometry and the value of *D*_active_. DTE molecules with *D*_active_ > 4.2 Å are not expected to be photoactive. However, the photoactivity of DTE molecules with *D*_active_ < 4.2 Å depends on the orientation of thio­phene rings. Thus, we attempted to combine *D*_active_ with two different vector-based parameters that represent the orientation of the thio­phene rings such as Φ_B_ [Fig. S5(*a*)]. Since enantiomeric pairs of any given DTE molecule have the same values but opposite signs, we were unable to unambiguously assign rotamers using them [Fig. S5(*b*)]. Therefore, the parameters for a given DTE conformer may be indistinguishable from those of the enantiomer of a different conformer [Fig. S5(*c*)].

**Figure 4:**
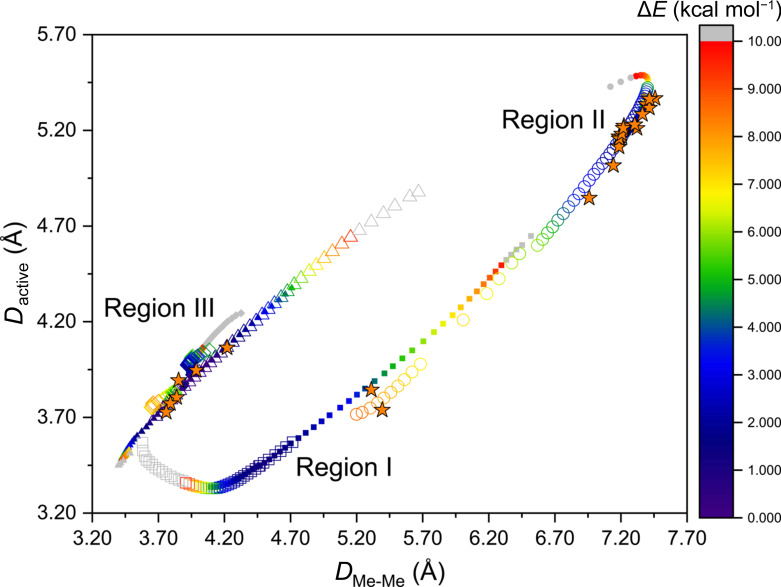
*D*_active_ versus *D*_Me-Me_. Filled shapes represent +1° increments, while empty shapes represent −1° increments. AP1 (square), AP2 (circle), P3 (diamond) and P4 (triangle), Experimental (orange star). Heatmap represents the relative energy Δ*E* (kcal mol^−1^).

The use of scalar-based parameters, namely interatomic distances, eliminates complications arising from the sign of the relative orientation of vectors. Likewise, these values are invariant to structure inversion. In other words, enantiomeric pairs will possess identical values. Therefore, we combined *D*_active_ with another scalar-based parameter *D*_Me-Me_ to describe the photoactive nature of DTE molecules. When used together, these two parameters describe how the thio­phene rings are orientated relative to each other. The plot of *D*_Me-Me_ versus *D*_active_ for the calculated DTE geometries takes on the shape of a broken ellipse (Fig. 4 ▸).

The lowest-energy calculated structures (Δ*E* < 5 kcal mol^−1^) for each of the conformers fall into one of three distinct regions. Region I, which contains the lowest-energy AP1 conformers, is characterized by *D*_active_ < *D*_Me-Me_ and *D*_active_ < 4.20 Å. Region II, where *D*_active_ > *D*_Me-Me_ and *D*_active_ > 4.20 Å, contains the AP2 conformers. Region III, where *D*_active_ ≃ *D*_Me-Me_ and *D*_active_ < 5.0 Å, comprises the P3/P4 conformers. Some overlap between regions does exist for the calculated structures. For example, the calculated structures for the two antiparallel conformers are present where 3.70 Å < *D*_active_ < 4.70 Å and 5.20 Å < *D*_Me-Me_ < 6.70 Å. However, this overlap only occurs for high-energy structures of a given conformer (Δ*E* > 5 kcal mol^−1^), and the geometries for which are unlikely to be observed in a crystalline solid.

*D*–*D* analysis (*D*_active_–*D*_Me-Me_ analysis) of the crystal structures confirmed that the experimental DTE geometries were clustered into the three regions described above. As anticipated, we found that the structures containing the AP1, AP2 and P3/P4 conformers are exclusively located in regions I, II and III, respectively. In fact, it is notable that most of the experimental structures are observed near the lowest-energy calculated structures. Though crystal packing forces may lead to slight deviations from the calculated minimum structures, the observed structures agree well with the minimum energy structures for each conformer type.

## 4. Conclusions

We found that 17 of the 19 observed DTE crystal structures (89.5%) contained a photoinactive conformer. Of these, 13 included the AP2 rotamer and 4 included the P3/P4 rotamers. The photoactive AP1 rotamer was only observed in 2 of the 19 crystal structures (10.5%). These results highlight the challenges associated with the design and synthesis of functional DTE-based crystalline materials. These challenges can be applied more broadly to any conformationally flexible system in which a specific conformation is desired in the crystalline phase. In the present case, the desired photoactivity is directly linked to the DTE conformation, which is not easily controlled in the crystalline state.

Future work will include higher level computational methods on gas phase and solvated DTE to improve the accuracy of rotamer energies. Periodic calculations using experimental crystal structures will be challenging, as these systems are large and complex. However, these calculations could provide valuable insight into the non-covalent interactions responsible for the stabilization of a given conformer within a structure.

The *D*–*D* analysis presented herein provides a rapid, effective and intuitive means of relating experimental and computational DTE geometries for the construction of the crystal landscape. The determination of the crystal landscape for this DTE, and more broadly for all di­aryl­ethenes, will provide structural insights that will link the supramolecular interactions found in crystalline solids to the observed conformers, and thus provide a basis for the rational design of next-generation photoactive crystalline materials. Crystal structure landscape analyses based on important geometric parameters and other key properties of interest will serve as an indispensable tool for the broader crystal engineering community.

## 5. Related literature

The following references are cited in the supporting information: Sheldrick (2008 ▸, 2015*a* ▸,*b* ▸); Dolomanov *et al.* (2009 ▸); Bruker (2013 ▸); Müller *et al.* (2006 ▸).

## Supplementary Material

Crystal structure: contains datablock(s) dte-1, dte-2, dte-3, dte-4, dte-5, dte-6, dte-7, dte-8, dte-9, dte-10, dte-11, dte-12, dte-13, dte-14, dte-15, dte-16, dte-17, dte-18, dte-19. DOI: 10.1107/S2052252523008060/yc5044sup1.cif

Structure factors: contains datablock(s) dte-1. DOI: 10.1107/S2052252523008060/yc5044dte-1sup2.hkl

Structure factors: contains datablock(s) dte-2. DOI: 10.1107/S2052252523008060/yc5044dte-2sup3.hkl

Structure factors: contains datablock(s) dte-3. DOI: 10.1107/S2052252523008060/yc5044dte-3sup4.hkl

Structure factors: contains datablock(s) dte-4. DOI: 10.1107/S2052252523008060/yc5044dte-4sup5.hkl

Structure factors: contains datablock(s) dte-5. DOI: 10.1107/S2052252523008060/yc5044dte-5sup6.hkl

Structure factors: contains datablock(s) dte-6. DOI: 10.1107/S2052252523008060/yc5044dte-6sup7.hkl

Structure factors: contains datablock(s) dte-7. DOI: 10.1107/S2052252523008060/yc5044dte-7sup8.hkl

Structure factors: contains datablock(s) dte-8. DOI: 10.1107/S2052252523008060/yc5044dte-8sup9.hkl

Structure factors: contains datablock(s) dte-9. DOI: 10.1107/S2052252523008060/yc5044dte-9sup10.hkl

Structure factors: contains datablock(s) dte-10. DOI: 10.1107/S2052252523008060/yc5044dte-10sup11.hkl

Structure factors: contains datablock(s) dte-11. DOI: 10.1107/S2052252523008060/yc5044dte-11sup12.hkl

Structure factors: contains datablock(s) dte-12. DOI: 10.1107/S2052252523008060/yc5044dte-12sup13.hkl

Structure factors: contains datablock(s) dte-13. DOI: 10.1107/S2052252523008060/yc5044dte-13sup14.hkl

Structure factors: contains datablock(s) dte-14. DOI: 10.1107/S2052252523008060/yc5044dte-14sup15.hkl

Structure factors: contains datablock(s) dte-15. DOI: 10.1107/S2052252523008060/yc5044dte-15sup16.hkl

Structure factors: contains datablock(s) dte-16. DOI: 10.1107/S2052252523008060/yc5044dte-16sup17.hkl

Structure factors: contains datablock(s) dte-17. DOI: 10.1107/S2052252523008060/yc5044dte-17sup18.hkl

Structure factors: contains datablock(s) dte-18. DOI: 10.1107/S2052252523008060/yc5044dte-18sup19.hkl

Structure factors: contains datablock(s) dte-19. DOI: 10.1107/S2052252523008060/yc5044dte-19sup20.hkl

Supporting data, figures and tables. DOI: 10.1107/S2052252523008060/yc5044sup21.pdf

CCDC references: 2162285, 2162286, 2162287, 2162288, 2162289, 2162290, 2162291, 2162292, 2162293, 2162294, 2162295, 2162296, 2162297, 2162298, 2162299, 2162300, 2162301, 2162302, 2162303

## Figures and Tables

**Table 1:** Relative energy (Δ*E*) and selected structural parameters for DTE RPES scans

Conformer	Δ*E* (kcal mol^−1^)	Φ_A_ (°)	Φ_B_ (°)	*D*_active_ (Å)	*D*_Me-Me_ (Å)
**(+)DTE-AP1**	1.11	51.46	173.76	3.45	4.47
**(+)DTE-AP2**	2.04	127.46	−84.97	5.19	7.25
**(+)DTE-P3**	0.00	−133.54	−29.77	3.87	3.91
**(+)DTE-P4**	0.04	−55.54	30.42	3.86	3.90
**(−)DTE-AP1**	1.11	51.46	173.76	3.45	4.47
**(−)DTE-AP2**	1.89	132.46	−94.49	5.18	7.22
**(−)DTE-P3**	0.00	−133.54	−29.91	3.87	3.91
**(−)DTE-P4**	1.87	−54.54	37.95	3.97	3.92
