# Supplementary material for: Development of a scalar-based geometric parameterization approach for the crystal structure landscape of dithienylethene-based crystalline solids
Source: IUCrJ. 2023 Sep 26;10(Pt 6):694–9. doi: 10.1107/S2052252523008060 (PMC10619447; doi:10.1107/S2052252523008060)
Supplement: Supplementary file 21 [file m-10-00694-sup21.pdf]

IUCrJ

Volume 10 (2023)

Supporting information for article:

Development of a Scalar-Based Geometric Parameterization Approach for the Crystal Structure Landscape of Dithienylethene-Based Crystalline Solids

Travis B. Mitchell, Xiaotong Zhang, Ronald T. Jerozal, Yu-Sheng Chen, SuYin Wang and Jason B. Benedict

S1. List of Acronyms Used:

- DTE – Dithienylethene
- **DTE** – (Z)-1,2-bis(2-methyl-5-(pyridin-4-yl)thiophen-3-yl)-1,2-diphenylethene
- SCXRD – Single crystal X-ray diffraction
- CDCl3 – Chloroform-*d*
- DFT – Density functional theory
- RPES – Relaxed potential energy surface
- CW – Clockwise
- CCW – Counter-clockwise
- **AP1** – Antiparallel active conformer
- **AP2** – Antiparallel inactive conformer
- **P3**/**P4** – Parallel conformer
- $Cu(OAc)_2$ – copper(II) acetate
- Cy_3PHBF_4 – tricyclohexylphosphonium tetrafluoroborate
- MeOH – methanol
- EtOH – ethanol
- PhMe – toluene
- B_2Pin_2 – bis(pinacolato)diboron
- EtOAc – ethyl acetate
- DCM – DCM
- NBS – N-bromosuccinimide
- $CHCl_3$ – chloroform
- AcOH – glacial acetic acid
- NaOH – sodium hydroxide
- $Na_2S_2O_3$ – sodium thiosulfate
- $MgSO_4$ – magnesium sulfate

- $Pd(PPh_3)_4$ – tetrakis(triphenylphosphine)palladium(0)
- K_2CO_3 – potassium carbonate
- K_3PO_4 – potassium tribasicphosphate
- DMF – N,N-dimethylformamide
- Et_2O – diethylether
- $Zn(NO_3)_2{\cdot}6H_2O$ – Zinc nitrate hexahydrate
- $Co(NO_3)_2{\cdot}6H_2O$ – Cobalt (II) nitrate hexahydrate
- $Ni(NO_3)_2{\cdot}6H_2O$ – Nickel (II) nitrate hexahydrate
- $Cd(NO_3)_2{\cdot}4H_2O$ – Cadmium nitrate tetrahydrate
- CuI – Copper (I) iodide
- $CuBr_2$ – Copper (II) bromide
- 5-NIP – 5-nitroisophthalic acid
- H_2OBA – 4,4'-oxybisbenzoic acid
- DMA - N,N-dimethylacetamide
- BPDC – biphenyl-4,4'-dicarboxylic acid
- **BMeDTS** – Methyl analogue of **DTE**

S2. Synthetic Details

S2.1. Molecular Synthesis

(Z)-1,2-diphenyl-1,2-bis(4,4,5,5-tetramethyl-1,3,2-dioxaborolan-2-yl)ethene (**3**): $Cu(OAc)_2$ (80.4 mg, 400 µmol), Cy_3PHBF_4 (520.6 mg, 1.4 mmol), MeOH (20 mL), and PhMe (6 mL) were added to a flame-dried 100 mL Schlenk flask. The reaction was allowed to run at 80 °C for 1 hour after which the reaction was cooled to room temperature, and the solvent was removed under reduced pressure. 1,2-diphenylethyne (3.5715 g, 20 mmol), B_2Pin_2 (6.6214 g, 26 mmol), and PhMe (6 mL) were added to the flask. The reaction was under a nitrogen atmosphere and stirred for 24 hours at 80 °C after which the reaction was cooled to room temperature and diluted with EtOAc (20 mL) and DCM (20 mL) and transferred to a 100 mL round bottom flask. The solvent was removed using a rotary evaporator. The crude product was purified using silica gel chromatography (1:1 DCM:Hexanes). The

product was a white solid (6.5059 g, 75%). ^{1}H NMR (400 MHz, $CDCl_3$) δ 7.10 – 6.99 (m, 6H), 6.98 – 6.89 (m, 4H), 1.32 (s, 24H).

3,5-dibromo-2-methylthiophene (**5**): NBS (35.6097 g, 80 mmol), $CHCl_3$ (100 mL), and AcOH (100 mL) were added to a 1000 mL round bottom flask equipped with a stir bar. 2-methylthiophene (9.8354 g, 100 mmol) was added to a 125 mL addition funnel with 20 mL of AcOH. The mixture was added over 30 minutes to the 500 mL round bottom flask. The round bottom flask was wrapped in aluminium foil and stirred for 24 hours. The reaction was poured over 200 mL of water and the organic phase was extracted with 50mL of DCM, washed with NaOH (200 mL, 1M), $Na_2S_2O_3$ (200 mL, 1M), brine, and dried over anhydrous $MgSO_4$. The solvent was removed using a rotary evaporator producing a dark red solution. The crude product was purified using silica gel chromatography (100% hexanes). The product was an off-yellow liquid (23.0909 g, 90%). ^{1}H NMR (400 MHz, $CDCl_3$) δ 6.84 (s, 1H), 2.32 (s, 3H).

4-(4-bromo-5-methylthiophen-2-yl)pyridine (**6**): 4-pyridylboronic acid (4.9412 g, 40 mmol), K_2CO_3 (10.5834 g, 80 mmol), and $Pd(PPh_3)_4$ (469.3 mg, 1 mol%) were added to a 500 mL round bottom Schlenk flask equipped with a stir bar. After the addition of each solid, the flask was evacuated and refilled with nitrogen gas three times. **1** (20.5062 g, 80 mmol), PhMe (160 mL), H_2O (45 mL), n-butanol (15 mL), and a few drops of Aliquat 336 were added to the Schlenk flask via syringe. The reaction was run under a nitrogen atmosphere and stirred for 24 hours at 70 °C. The reaction was cooled to room temperature and diluted with water (50 mL) and EtOAc (50 mL). The organic phase was separated, washed with water (2x300 mL), brine (2x300 mL), and dried over anhydrous $MgSO_4$. The solvent was removed using a rotary evaporator. The crude product was purified using silica gel chromatography (30% EtOAc in hexanes to 40% EtOAc in hexanes) The product was a light-yellow solid. (4.6457 g, 45%). ^{1}H NMR (400 MHz, $CDCl_3$) δ 8.58 (t, J = 6.3, 4.4 Hz, 2H), 7.40 (dd, J = 6.3, 4.3 Hz, 2H), 7.33 (s, 1H), 2.45 (s, 3H).

(Z)-1,2-bis(2-methyl-5-(pyridin-4-yl)thiophen-3-yl)-1,2-diphenylethene (**DTE**): **2** (4.0791 g, 16 mmol), 3 (3.6484 g, 8 mmol), K_3PO_4 (10.2551 g, 48 mmol), and $Pd(PPH_3)_4$ (492.3 mg, 400 μmol), were added to a 250 mL Schlenk tube. After the addition of each solid, the Schlenk tube was evacuated and refilled with nitrogen gas three times. PhMe (56 mL), water (16 mL), n-butanol (6 mL), and a few drops of Aliquat 336 were added to the Schlenk tube via syringe. The reaction was run under a nitrogen atmosphere and stirred for 48 hours at 100 °C. The reaction was cooled to room temperature and diluted with water (30 mL) and EtOAc (30 mL). the organic phase was separated, washed with water (2x300 mL), brine (2x300 mL), and the organic phase was further dried over anhydrous $MgSO_4$. The solvent was removed using a rotary evaporator. The crude product was purified using silica gel chromatography (50% EtOAc in Hexanes to 100% EtOAc). The product was a yellow solid. DTE was recrystallized from a solution of EtOAc producing block crystals (**DTE-1**).

(409.1 mg, 10%). ^{1}H NMR (500 MHz, $CDCl_3$) δ 8.47 (d, J = 5.3 Hz, 4H), 7.26 – 7.21 (m, 4H), 7.18 – 7.10 (m, 6H), 7.09 – 7.03 (m, 4H), 7.01 (s, 2H), 2.05 (s, 6H).

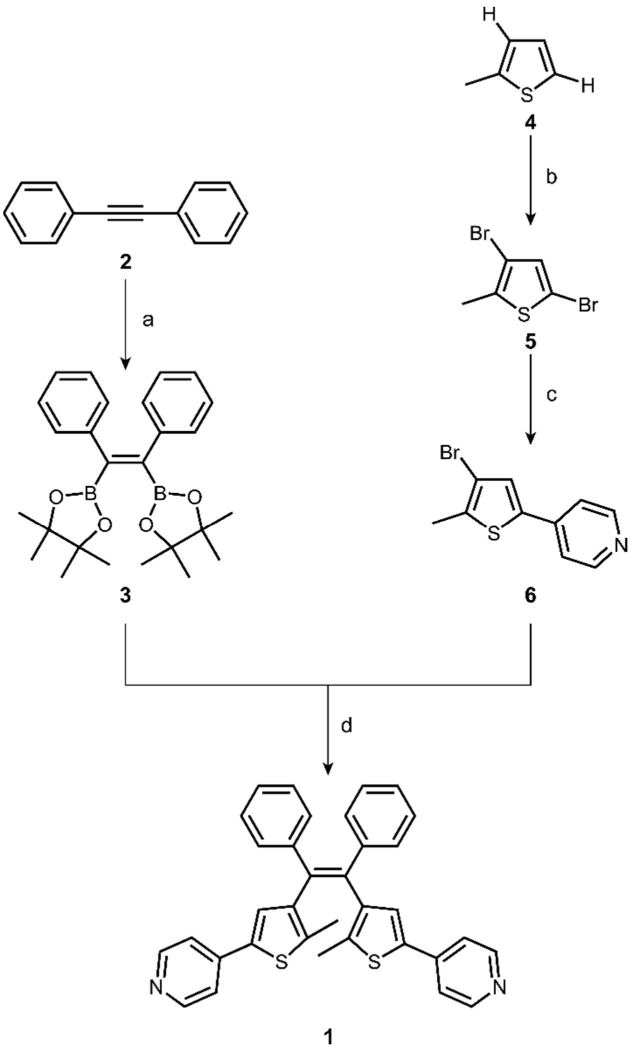

Figure S1 Synthetic route of **DTE**. (a) $Cu(OAc)_2$, CY_3PHBF_4, B_2Pin_2, MeOH, PhMe, 70 °C, 24 h, 75%. (b) NBS, $CHCl_3$, AcOH, RT, 24 h, 90%. (c) 4-pyridylboronic acid, $Pd(PPh_3)_4$, K_3PO_4, H_2O, PhMe, n-Butanol, Aliquat 336, 70 °C, 24 h, 45%. (d) 4-pyridylboronic acid, $Pd(PPh_3)_4$, K_3PO_4, H_2O, PhMe, n-Butanol, Aliquat 336, 100 °C, 48 h, 10%.

S2.2. Single Crystal Synthesis

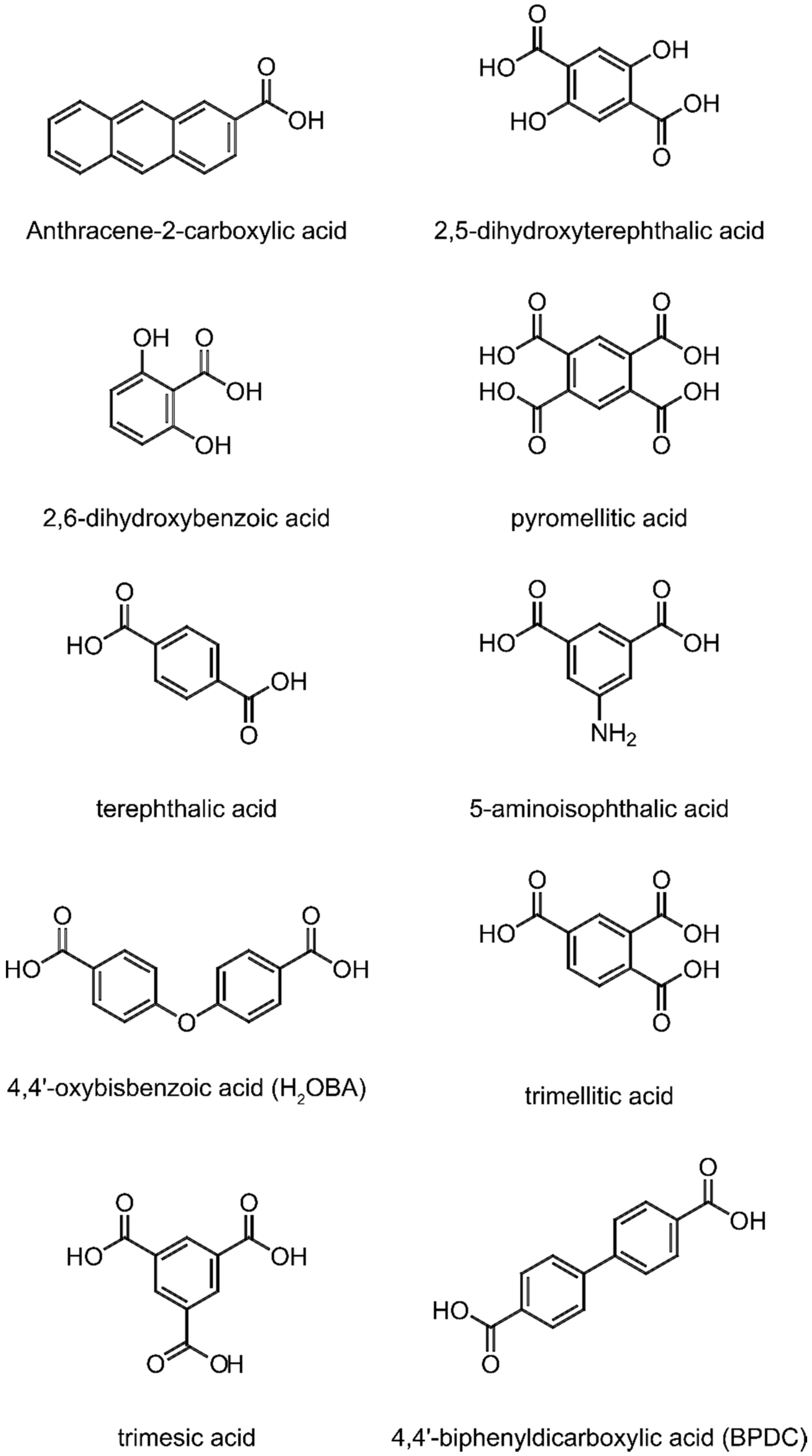

Figure S2 Chemical structures of the linkers and co-formers used in the crystal syntheses.

DTE-2: **DTE** (10.3 mg, 19 µmol), Anthracene-2-carboxylic acid (5.7 mg), $Zn(NO_3)_2{\cdot}6H_2O$ (5.4 mg, 19 µmol), DMF (1 mL), and H_2O (1 mL) were added to a 15 mL thick-walled vial. The thick-walled vial was capped and placed in a pre-heated oven set to 100 °C. After 24 Hours, the thick-walled vial was removed from the oven and cooled to room temperature yielding colourless prisms.

DTE-3: **DTE** (5.4 mg, 9.5 µmol), 2,5-dihydroxyterephthalic acid (1.9 mg, 9.5 µmol), and MeOH (5 mL) were gently heated in a 25 mL scintillation vial. The clear-colourless solution was allowed to slowly evaporate in the dark producing yellow prisms.

DTE-4: **DTE** (10.4 mg, 19 µmol), 2,6-dihydroxybenzoic acid (6.4 mg, 38 µmol), and MeOH (5 mL) were gently heated in a 25 mL scintillation vial. The clear-colourless solution was allowed to slowly evaporate in the dark producing yellow plates.

DTE-5: **DTE** (5.0 mg, 9.49 µmol), pyromellitic acid (4.8 mg 18.98 µmol), and EtOH (10 mL) were gently heated in a 25 mL scintillation vial. The clear-colourless solution was allowed to slowly evaporate in the dark producing green prisms.

DTE-6: **DTE** (10.4 mg, 19 µmol), terephthalic acid (3.7 mg, 19 µmol), and MeOH (5 mL) were gently heated in a 25 mL scintillation vial. The solution was filtered, and the clear-colourless solution was allowed to slowly evaporate in the dark producing yellow plates.

DTE-7: **DTE** (9.7 mg, 19 µmol), $Co(NO_3)_2{\cdot}6H_2O$ (5.4 mg, 19 µmol), MeOH (1 mL), and ACN (1mL), and DCM (1 mL) were added to a 25 mL scintillation vial. The pale-pink solution was allowed to evaporate slowly in the dark producing orange prisms.

DTE-8: **DTE** (10.3 mg, 20 µmol), $Ni(NO_3)_2{\cdot}6H_2O$ (5.4 mg, 19 µmol), methanol (1 mL), and ACN (1mL), and DCM (1 mL) were added to a 25 mL scintillation vial. The pale-green solution was allowed to evaporate slowly in the dark producing light-green prisms.

DTE-9: **DTE** (11.3 mg, 21 µmol), $Zn(NO_3)_2{\cdot}6H_2O$ (7.5 mg, 25 µmol), methanol (1 mL), and ACN (1mL), and DCM (1 mL) were added to a 25 mL scintillation vial. The colourless solution was allowed to evaporate slowly in the dark producing colourless prisms.

DTE-10: **DTE** (9.7 mg, 18 µmol), $Cd(NO_3)_2{\cdot}4H_2O$ (5.7 mg, 18 µmol), methanol (1 mL), and ACN (1mL), and DCM (1 mL) were added to a 25 mL scintillation vial. The colourless solution was allowed to evaporate slowly in the dark producing colourless prisms.

DTE-11: **DTE** (11.0 mg, 21 µmol), CuI (4.3 mg, 23 µmol), MeOH (1 mL), and ACN (1mL), and DCM (1 mL) were added to a 25 mL scintillation vial. The grey-turbid solution was allowed to evaporate slowly in the dark producing yellow plates.

DTE-12: **DTE** (10.3 mg, 20 µmol), $CuBr_2$ (5.4 mg, 24 µmol), and DMF (1 mL) were added to a 1-Dram vial producing a dark green solution. This uncapped vial was placed in a 25 mL scintillation vial with Et_2O (1 mL). The 25 mL scintillation vial was capped, and the Et_2O vapours were allowed to diffuse into the green DMF solution overnight precipitating out green prisms.

DTE-13: **DTE** (4.8 mg, 9 µmol), 5-NIP (2.1 mg, 10 µmol), $Zn(NO_3)_2{\cdot}6H_2O$ (2.4 mg, 8 µmol), DMF (0.5 mL), and H_2O (0.5 mL) were added to a 15 mL thick-walled vial. The thick-walled vial was

capped and placed in a pre-heated oven set to 90 °C. After 24 Hours, the thick-walled vial was removed from the oven and cooled to room temperature to yield colourless plates.

DTE-14: **DTE** (20.1 mg, 38 µmol), H_2OBA (9.9 mg, 38 µmol), $Zn(NO_3)_2{\cdot}6H_2O$ (12.0 mg, 40 µmol), DMF (1 mL), and H_2O (1 mL) were added to a 15 mL thick-walled vial. The thick-walled vial was capped and placed in a pre-heated oven set to 90 °C. After 72 Hours, the thick-walled vial was removed from the oven and cooled to room temperature to yield colourless plates.

DTE-15: **DTE** (5.3 mg, 10 µmol), Pyromelliltic Acid (3.0 mg, 12 µmol), $Zn(NO_3)_2{*}6H_2O$ (3.8 mg, 13 µmol), DMA(0.5 mL), and H_2O (0.5 mL) were added to a 15 mL thick-walled vial. The thick-walled vial was capped and placed in a pre-heated oven set to 100 °C. After 24 hours, the thick-walled vial was removed from the oven and cooled to room temperature to yield colourless prisms.

DTE-16: **DTE** (10.0 mg, 19 µmol), Trimellitic Acid (4.0 mg, 19 µmol), $Zn(NO_3)_2{\cdot}6H_2O$ (5.6 mg, 19 µmol), DMF (1 mL), and H_2O (1 mL) were added to a 15 mL thick-walled vial. The thick-walled vial was capped and placed in a pre-heated oven set to 90 °C. After 24 Hours, the thick-walled vial was removed from the oven and cooled to room temperature to yield colourless prisms.

DTE-17: **DTE** (10.2 mg, 19 µmol), Trimesic Acid (4.6 mg, 22 µmol), $Zn(NO_3)_2{*}6H_2O$ (6.0 mg, 19 µmol), DMF (1 mL), and H_2O (1 mL) were added to a 15 mL thick-walled vial. The thick-walled vial was capped and placed in a pre-heated oven set to 90 °C. After 24 Hours, the thick-walled vial was removed from the oven and cooled to room temperature to yield colourless prisms.

DTE-18: **DTE** (10.7 mg, 20 µmol), BPDC (5.6 mg, 23 µmol), $Zn(NO_3)_2{*}6H_2O$ (5.9 mg, 19 µmol), DMF (2 mL) were added to a 15 mL thick-walled vial. The thick-walled vial was capped and placed in a pre-heated oven set to 130 °C. After 16 Hours, the thick-walled vial was removed from the oven and cooled to room temperature which initially yielded no crystals. After a year of constant monitoring, colourless prisms formed.

DTE-19: **DTE** (10.9 mg, 20 µmol), Terephthalic Acid (3.0 mg, 18 µmol), $Zn(NO_3)_2{*}6H_2O$ (11.9 mg, 40 µmol), DMF (0.40 mL), H_2O (0.75 mL), and MeOH (0.40 mL) were added to a 15 mL thick-walled vial. The thick-walled vial was capped and placed in a pre-heated oven set to 100 °C. After 24 Hours, the thick-walled vial was removed from the oven and cooled to room temperature which initially yielded no crystals. After a year of constant monitoring, colourless prisms formed.

S3. Crystallographic Details

S3.1. Synchrotron Source Data Collection

A single crystal suitable for X-ray diffraction was mounted on the tip of a glass fibre with oil and placed on a Huber three-circle diffractometer at the National Science Foundation's (NSF) ChemMatCARS beamline 15ID-D at Argonne National Laboratory (λ = 0.41328 Å). Using a Dectris

Pilatus3X 1M (CdTe) shutterless detector at 130 mm from the crystal, frames were collected with ω = -180° and a 2θ-angle of 0°. During the data collection, the crystal was cooled to 100 K using an Oxford cryojet nitrogen-gas flow apparatus. A total of 1440 frames were collected during two 360° φ-scans (0.5° image width) at κ = 45° and κ = 30°, nominally covering complete reciprocal space. Dectris frames (.cbf) were converted to Bruker format (.sfrm) using custom software developed by NSF's ChemMatCARS. Following frame conversion, indexing was performed using the Bruker APEX3 software suite.(Bruker, 2013)

S3.2. Home Source Data Collection

A single crystal suitable for X-ray diffraction was mounted on the tip of a glass fibre with oil and placed on a Bruker SMART APEX II CCD diffractometer installed at a rotating anode source (Mo-Kα radiation, λ = 0.71073 Å) with a detector distance of 40.00 mm from the crystal and a 2θ-angle of -30°. During the data collection, the crystal was cooled to 90(1) K using an Oxford cryostream nitrogen gas-flow apparatus. A total of 1800 frames were collected using five 180° ω-scans (0.5° scan width) at different φ-angles (φ = 0° to 288° in 72° increments), nominally covering complete reciprocal space.

S3.3. Data Reduction and Analysis

Data reduction was completed using SAINT version 8.40A, and a multi-scan absorption correction was applied using SADABS version 2016 included in the Bruker APEX3 software suite. Space-group determination was performed using the XPREP utility included in the SHELXTL software package.(Sheldrick, 2008) Using Olex2,(Dolomanov *et al.*, 2009) the structure was solved with ShelXT(Sheldrick, 2015a) using intrinsic phasing and refined with ShelXL(Sheldrick, 2015b) using least squares minimization (full-matrix least-squares on F^2). Hydrogen atoms bonded to oxygen and nitrogen atoms were located in the Fourier difference map. Their distances were restrained using the DFIX command with the recommended O-H distance of 0.84 Å and N-H distance of 0.88 Å found in the .lst file with $U_{ISO}(H) = 1.2U_{eq}$ (O or N).(Müller *et al.*, 2006)

Table S1 Selected geometric parameters for each crystallographically unique DTE.

Molecule ID	Conformation	Φ_A (°)	Φ_B (°)	D_{Active} (Å)	$D_{Me\text{-}Me}$ (Å)
**DTE-1**	**AP2**	126.10	-110.33	5.11	7.18
**DTE-2**	**AP2**	137.08	-95.11	5.33	7.39
**DTE-3A**	**AP2**	125.29	-99.70	5.02	7.14
**DTE-3B**		-132.83	99.76	5.21	7.32
**DTE-4A**	**P3/P4**	-130.45	-15.84	3.81	3.84
**DTE-4B**		-63.41	24.63	3.89	3.85
**DTE-5**	**P3/P4**	-140.48	-30.62	3.95	3.99
**DTE-6A**	**P3/P4**	-131.56	-23.83	3.77	3.79
**DTE-6B**		-61.81	23.69	3.73	3.76
**DTE-7**	**AP2**	131.55	-100.09	5.17	7.19
**DTE-8**	**AP2**	131.55	-101.21	5.17	7.20
**DTE-9**	**AP2**	131.37	-100.06	5.15	7.18
**DTE-10**	**AP2**	131.64	-98.09	5.18	7.22
**DTE-11**	**P3/P4**	-129.34	-48.22	4.06	4.22
**DTE-12**	**AP2**	133.05	-98.84	5.22	7.22
**DTE-13**	**AP2**	137.56	-89.43	5.36	7.46
**DTE-14A**	**AP2**	130.11	-108.90	5.21	7.22
**DTE-14B**		-122.99	124.44	4.85	6.96
**DTE-15A**	**AP2**	138.44	-89.93	5.32	7.41
**DTE-15B**		137.55	-92.24	5.28	7.36
**DTE-16**	**AP2**	134.16	-98.84	5.23	7.30
**DTE-17**	**AP2**	138.25	-95.11	5.35	7.41
**DTE-18**	**AP1**	75.06	-169.41	3.73	5.38
**DTE-19**	**AP1**	70.40	-169.41	3.85	5.31

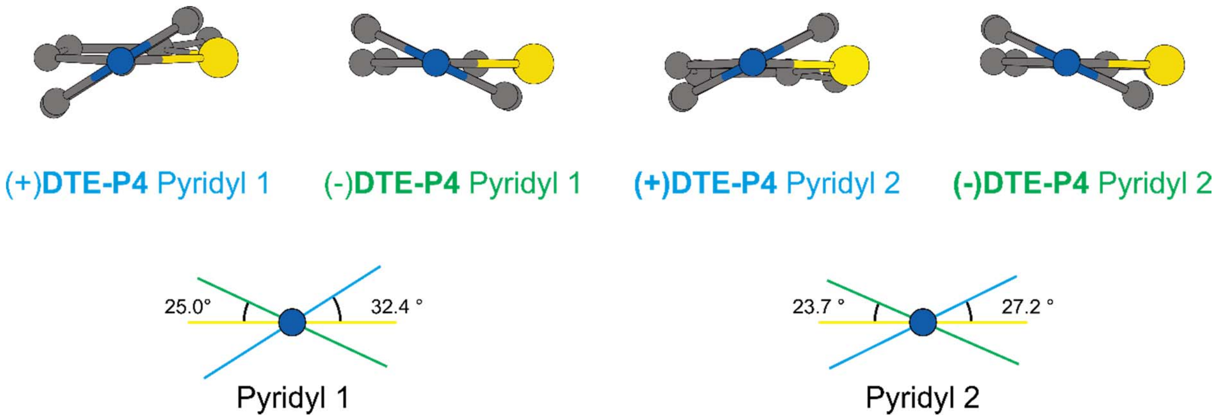

Figure S3 Difference in planarity of Pyridyl 1 and Pyridyl 2 for **DTE-P4** in each scan direction (positive, blue; negative, green).

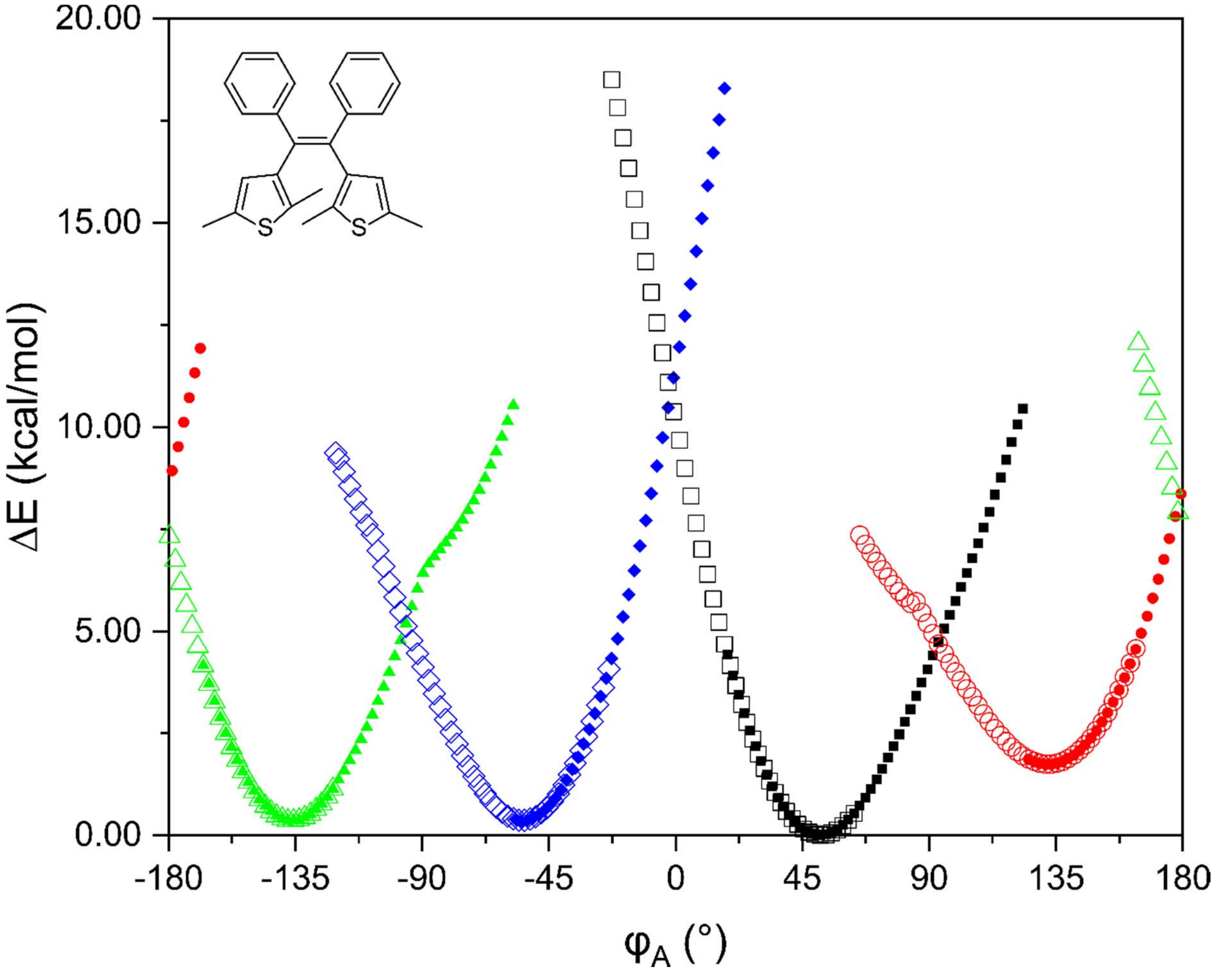

Figure S4 RPES plot for **BMeDTS**. Filled shapes represent +1° increments, while empty shapes represent -1° increments. **AP1** (black, square), **AP2** (red, circle), **P3** (green, triangle), and **P4** (blue, diamond).

Table S2 Relative energy (ΔE) and selected structural parameters for **BMeDTS**.

Conformer	Scan Direction	ΔE (kcal/mol)	φ_A (°)	φ_B (°)	D_{Active} (Å)	$D_{Me\text{-}Me}$ (Å)
**DTE-AP1**		0.00	51.34	172.35	3.46	4.44
**DTE-AP2**	+ 1	1.73	132.34	-102.57	5.19	7.20
**DTE-P3**		0.37	-135.66	-37.40	3.98	3.92
**DTE-P4**		0.36	-54.66	37.55	3.98	3.92
**DTE-AP1**		0.00	51.34	172.38	3.46	4.44
**DTE-AP2**	- 1	1.74	132.34	-102.64	5.19	7.20
**DTE-P3**		0.36	-136.66	-37.78	3.99	3.93
**DTE-P4**		0.37	-54.66	37.71	3.99	3.93

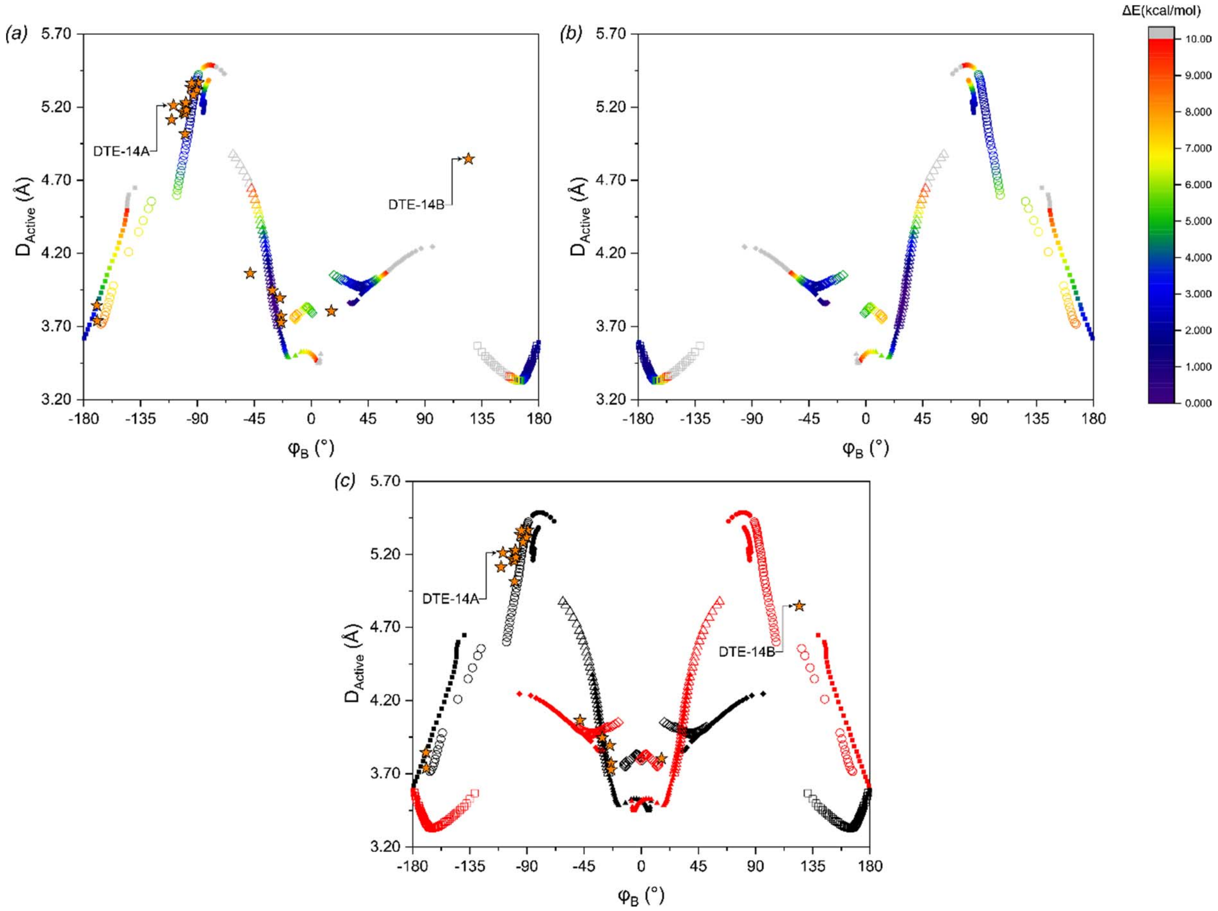

Figure S5 Plots of D_{active} vs. ϕ_B for **DTE** (*a*), the enantiomer of **DTE** (*b*) and the overlay of DTE (*c*, black) and its enantiomer (*c*, red). Filled shapes represent +1° increments, while empty shapes represent -1° increments. **AP1** (square), **AP2** (circle), **P3** (diamond), and **P4** (triangle), Experimental (orange star). Heatmap represents the relative energy (ΔE, kcal/mol).

S4. Crystallography Tables

Table S3 Crystal data and structure refinement for **DTE-[1-2]**.

Identification code	**DTE-[1]**	**DTE-[2]**
Empirical formula	$C_{34}H_{26}N_2S_2$	$C_{34}H_{26}N_2S_2$
Formula weight	526.69	526.69
Temperature (K)	90	90
Crystal system	triclinic	monoclinic
Space group	*P*-1	*I*2/*a*
a (Å)	9.4895(4)	9.8916(4)
b (Å)	11.6539(5)	25.9653(10)
c (Å)	13.4967(6)	10.9370(6)
α (°)	114.1250(10)	90
β (°)	100.2420(10)	109.5250(10)
γ (°)	91.8530(10)	90
Volume ($Å^3$)	1331.32(10)	2647.5(2)
Z	2	4
ρ_{calc} (g cm^{-3})	1.314	1.321
μ (mm^{-1})	0.227	0.228
F(000)	552	1104
Crystal size (mm^3)	0.06 × 0.04 × 0.04	0.2 × 0.04 × 0.02
Radiation	MoKα (λ = 0.71073)	MoKα (λ = 0.71073)
2Θ range for data collection (°)	3.382 to 56.566	3.138 to 66.368
Index ranges	$-12 \le h \le 12$, $-15 \le k \le 15$, $-17 \le l \le 17$	$-15 \le h \le 15$, $-39 \le k \le 39$, $-16 \le l \le 16$
Reflections collected	27314	28558
Independent reflections	6607 [R_{int} = 0.0439, R_{sigma} = 0.0421]	5068 [R_{int} = 0.0196, R_{sigma} = 0.0132]
Data/restraints/parameters	6607/0/347	5068/0/220
Goodness-of-fit on F^2	1.043	1.056
Final R indexes [I>=2σ (I)]	R_1 = 0.0439, wR_2 = 0.0963	R_1 = 0.0337, wR_2 = 0.0908
Final R indexes [all data]	R_1 = 0.0603, wR_2 = 0.1036	R_1 = 0.0372, wR_2 = 0.0944
Largest diff. peak/hole (e $Å^{-3}$)	0.47/-0.31	0.56/-0.26
Flack Parameter	N/A	N/A

Table S4 Crystal data and structure refinement for **DTE-[3-4].**

Identification code	**DTE-[3]**	**DTE-[4]**
Empirical formula	$C_{84}H_{64}N_4O_{12}S_4$	$C_{185}H_{145}N_8O_{28}S_8$
Formula weight	1449.63	3184.56
Temperature (K)	90	90
Crystal system	monoclinic	triclinic
Space group	*P*2/*n*	*P*-1
a (Å)	17.1862(6)	10.602(2)
b (Å)	12.2494(4)	16.714(4)
c (Å)	18.3174(6)	24.181(5)
α (°)	90	71.454(6)
β (°)	113.2730(10)	88.132(6)
γ (°)	90	78.607(6)
Volume ($Å^3$)	3542.4(2)	3980.4(14)
Z	2	1
ρ_{calc} (g cm^{-3})	1.359	1.329
μ (mm^{-1})	0.203	0.189
F(000)	1512	1663
Crystal size (mm^3)	0.2 × 0.1 × 0.04	0.16 × 0.14 × 0.04
Radiation	MoKα (λ = 0.71073)	MoKα (λ = 0.71073)
2Θ range for data collection (°)	3.324 to 61.014	2.622 to 49.424
Index ranges	-24 ≤ h ≤ 24, -17 ≤ k ≤ 17, -26 ≤ l ≤ 26	-11 ≤ h ≤ 12, -19 ≤ k ≤ 19, -28 ≤ l ≤ 28
Reflections collected	80900	37926
Independent reflections	10820 [R_{int} = 0.0585, R_{sigma} = 0.0368]	13567 [R_{int} = 0.0588, R_{sigma} = 0.0746]
Data/restraints/parameters	10820/7/496	13567/323/1176
Goodness-of-fit on F^2	1.022	1.095
Final R indexes [I>=2σ (I)]	R_1 = 0.0453, wR_2 = 0.1111	R_1 = 0.0879, wR_2 = 0.2205
Final R indexes [all data]	R_1 = 0.0622, wR_2 = 0.1225	R_1 = 0.1188, wR_2 = 0.2382
Largest diff. peak/hole (e $Å^{-3}$)	0.48/-0.49	1.60/-0.46
Flack Parameter	N/A	N/A

Table S5 Crystal data and structure refinement for **DTE-[5-6].**

Identification code	**DTE-[5]**	**DTE-[6]**
Empirical formula	$C_{49}H_{35}N_2O_{12}S_2$	$C_{42}H_{32}N_2O_4S_2$
Formula weight	907.91	692.81
Temperature (K)	90	90
Crystal system	monoclinic	orthorhombic
Space group	$P2_1/c$	$Pna2_1$
*a (*Å)	9.6987(2)	30.6739(12)
b (Å)	20.7245(5)	9.6686(4)
c (Å)	22.9341(6)	24.2850(9)
α (°)	90	90
β (°)	101.6800(10)	90
γ (°)	90	90
Volume (Å^3)	4514.32(19)	7202.3(5)
Z	4	8
ρ_{calc} (g cm^{-3})	1.336	1.278
μ (mm^{-1})	0.184	0.193
F(000)	1884	2896
Crystal size (mm^3)	0.12 × 0.06 × 0.04	0.2 × 0.16 × 0.02
Radiation	MoKα (λ = 0.71073)	MoKα (λ = 0.71073)
2Θ range for data collection (°)	2.674 to 52.802	2.656 to 61.014
Index ranges	$-10 \le h \le 12$, $-25 \le k \le 25$, $-28 \le l \le 28$	$-43 \le h \le 43$, $-13 \le k \le 13$, $-34 \le l \le 34$
Reflections collected	57175	162249
Independent reflections	9238 [R_{int} = 0.0789, R_{sigma} = 0.0641]	21979 [R_{int} = 0.0574, R_{sigma} = 0.0336]
Data/restraints/parameters	9238/5/631	21979/5/919
Goodness-of-fit on F^2	1.022	1.032
Final R indexes [I>=2σ (I)]	R_1 = 0.0519, wR_2 = 0.1034	R_1 = 0.0358, wR_2 = 0.0841
Final R indexes [all data]	R_1 = 0.0923, wR_2 = 0.1180	R_1 = 0.0433, wR_2 = 0.0880
Largest diff. peak/hole (e Å^{-3})	0.46/-0.40	0.28/-0.21
Flack Parameter	N/A	0.41(4)

Table S6 Crystal data and structure refinement for **DTE-[7-8].**

Identification code	**DTE-[7]**	**DTE-[8]**
Empirical formula	$C_{68}H_{56}CoN_4O_2S_4$	$C_{68}H_{56}N_4NiO_2S_4$
Formula weight	1148.33	1148.11
Temperature (K)	90	100
Crystal system	orthorhombic	orthorhombic
Space group	*Fddd*	*Fddd*
a (Å)	9.8404(4)	9.8621(6)
b (Å)	21.4458(8)	21.6164(13)
c (Å)	67.537(3)	67.069(4)
α (°)	90	90
β (°)	90	90
γ (°)	90	90
Volume (Å^3)	14252.6(10)	14298.1(15)
Z	8	8
ρ_{calc} (g cm^{-3})	1.07	1.067
μ (mm^{-1})	0.398	0.106
F(000)	4792	4800
Crystal size (mm^3)	0.06 × 0.06 × 0.02	0.04 × 0.02 × 0.02
Radiation	MoKα (λ = 0.71073)	synchrotron (λ = 0.41328)
2Θ range for data collection (°)	3.986 to 56.602	2.662 to 28.134
Index ranges	$-13 \le h \le 13$, $-28 \le k \le 27$, $-90 \le l \le 54$	$-11 \le h \le 11$, $-24 \le k \le 24$, $-78 \le l \le 78$
Reflections collected	29065	71940
Independent reflections	4446 [R_{int} = 0.0468, R_{sigma} = 0.0347]	3003 [R_{int} = 0.0604, R_{sigma} = 0.0297]
Data/restraints/parameters	4446/0/182	3003/0/182
Goodness-of-fit on F^2	1.034	1.086
Final R indexes [I>=2σ (I)]	R_1 = 0.0480, wR_2 = 0.1055	R_1 = 0.0378, wR_2 = 0.0977
Final R indexes [all data]	R_1 = 0.0663, wR_2 = 0.1143	R_1 = 0.0435, wR_2 = 0.1011
Largest diff. peak/hole (e Å^{-3})	0.58/-0.66	0.24/-0.35
Flack Parameter	N/A	N/A

Table S7 Crystal data and structure refinement for **DTE-[9-10].**

Identification code	**DTE-[9]**	**DTE-[10]**
Empirical formula	$C_{68}H_{56}N_6O_8S_4Zn$	$C_{68}H_{56}CdN_6O_8S_4$
Formula weight	1278.79	1325.82
Temperature (K)	90	90
Crystal system	orthorhombic	orthorhombic
Space group	*Fddd*	*Fddd*
a (Å)	9.8296(5)	9.8116(12)
b (Å)	21.4844(12)	21.432(3)
c (Å)	67.400(4)	69.162(8)
α (°)	90	90
β (°)	90	90
γ (°)	90	90
Volume (Å^3)	14233.7(13)	14543(3)
Z	8	8
ρ_{calc} (g cm^{-3})	1.193	1.211
μ (mm^{-1})	0.516	0.468
F(000)	5312	5456
Crystal size (mm^3)	0.16 × 0.16 × 0.02	0.14 × 0.14 × 0.02
Radiation	MoKα (λ = 0.71073)	MoKα (λ = 0.71073)
2Θ range for data collection (°)	3.98 to 61.17	3.98 to 56.662
Index ranges	-13 ≤ h ≤ 14, -30 ≤ k ≤ 30, -96 ≤ l ≤ 75	-13 ≤ h ≤ 13, -28 ≤ k ≤ 28, -92 ≤ l ≤ 92
Reflections collected	47600	66341
Independent reflections	5466 [R_{int} = 0.0481, R_{sigma} = 0.0290]	4548 [R_{int} = 0.0476, R_{sigma} = 0.0159]
Data/restraints/parameters	5466/86/243	4548/47/220
Goodness-of-fit on F^2	1.21	1.268
Final R indexes [I>=2σ (I)]	R_1 = 0.0863, wR_2 = 0.2209	R_1 = 0.0927, wR_2 = 0.2321
Final R indexes [all data]	R_1 = 0.0965, wR_2 = 0.2268	R_1 = 0.0937, wR_2 = 0.2326
Largest diff. peak/hole (e Å^{-3})	1.09/-0.50	1.41/-0.86
Flack Parameter	N/A	N/A

Table S8 Crystal data and structure refinement for **DTE-[11-12].**

Identification code	**DTE-[11]**	**DTE-[12]**
Empirical formula	$C_{35}H_{28}Cl_2CuIN_2S_2$	$C_{74}H_{66}Br_2CuN_6O_2S_4$
Formula weight	802.05	1422.92
Temperature (K)	90	90
Crystal system	triclinic	triclinic
Space group	*P*-1	*P*-1
*a (*Å)	9.496(3)	9.5108(3)
b (Å)	10.032(3)	11.4444(3)
c (Å)	17.380(5)	16.0164(4)
α (°)	86.772(8)	105.0120(10)
β (°)	84.163(9)	98.4520(10)
γ (°)	83.443(9)	99.1780(10)
Volume (Å^3)	1634.7(8)	1629.52(8)
Z	2	1
ρ_{calc} (g cm^{-3})	1.629	1.45
μ (mm^{-1})	1.932	1.742
F(000)	800	731
Crystal size (mm^3)	0.14 × 0.06 × 0.01	0.6 × 0.4 × 0.4
Radiation	MoKα (λ = 0.71073)	MoKα (λ = 0.71073)
2Θ range for data collection (°)	4.092 to 49.426	2.686 to 66.406
Index ranges	-11 ≤ h ≤ 11, -11 ≤ k ≤ 11, -20 ≤ l ≤ 20	-14 ≤ h ≤ 14, -17 ≤ k ≤ 17, -24 ≤ l ≤ 24
Reflections collected	15927	44606
Independent reflections	5512 [R_{int} = 0.0697, R_{sigma} = 0.0926]	12487 [R_{int} = 0.0331, R_{sigma} = 0.0341]
Data/restraints/parameters	5512/0/392	12487/114/454
Goodness-of-fit on F^2	1.046	1.051
Final R indexes [I>=2σ (I)]	R_1 = 0.0553, wR_2 = 0.1279	R_1 = 0.0440, wR_2 = 0.1104
Final R indexes [all data]	R_1 = 0.0844, wR_2 = 0.1388	R_1 = 0.0580, wR_2 = 0.1167
Largest diff. peak/hole (e Å^{-3})	1.56/-0.99	1.70/-0.71
Flack Parameter	N/A	N/A

Table S9 Crystal data and structure refinement for **DTE-[13-14].**

Identification code	**DTE-[13]**	**DTE-[14]**
Empirical formula	$C_{42}H_{33}N_3O_8S_2Zn$	$C_{82}H_{60}N_4O_5S_4Zn$
Formula weight	837.2	1374.95
Temperature (K)	90	90
Crystal system	triclinic	triclinic
Space group	*P*-1	*P*-1
a (Å)	10.1753(13)	14.2712(9)
b (Å)	10.9520(13)	14.7916(9)
c (Å)	18.411(2)	19.2839(12)
α (°)	89.839(4)	98.124(2)
β (°)	82.711(4)	104.400(2)
γ (°)	69.145(4)	117.968(2)
Volume (Å^3)	1899.8(4)	3320.3(4)
Z	2	2
ρ_{calc} (g cm^{-3})	1.464	1.375
μ (mm^{-1})	0.815	0.555
F(000)	864	1428
Crystal size (mm^3)	0.14 × 0.1 × 0.02	0.14 × 0.02 × 0.01
Radiation	MoKα (λ = 0.71073)	MoKα (λ = 0.71073)
2Θ range for data collection (°)	3.984 to 56.694	3.27 to 56.564
Index ranges	$-13 \leq h \leq 13$, $-14 \leq k \leq 14$, $0 \leq l \leq 24$	$-19 \leq h \leq 18$, $-17 \leq k \leq 19$, $-25 \leq l \leq 19$
Reflections collected	11887	41065
Independent reflections	11887 [R_{int} = 0.0533, R_{sigma} = 0.0518]	16450 [R_{int} = 0.0467, R_{sigma} = 0.0693]
Data/restraints/parameters	11887/0/514	16450/0/873
Goodness-of-fit on F^2	1.053	1.014
Final R indexes [I>=2σ (I)]	R_1 = 0.0428, wR_2 = 0.1027	R_1 = 0.0446, wR_2 = 0.0888
Final R indexes [all data]	R_1 = 0.0508, wR_2 = 0.1060	R_1 = 0.0744, wR_2 = 0.0991
Largest diff. peak/hole (e Å^{-3})	0.54/-0.68	0.46/-0.48
Flack Parameter	N/A	N/A

Table S10 Crystal data and structure refinement for **DTE-[15-16].**

Identification code	DTE-[15]	DTE-[16]
Empirical formula	C90H93N7O17S4Zn2	C46H39N3O8S2Zn
Formula weight	1803.69	891.29
Temperature (K)	90	90
Crystal system	triclinic	triclinic
Space group	*P*-1	*P*-1
a (Å)	11.873(2)	10.1309(4)
b (Å)	17.012(3)	11.6998(4)
c (Å)	22.921(4)	19.7642(7)
α (°)	78.006(4)	76.4910(10)
β (°)	76.999(4)	78.6250(10)
γ (°)	83.799(4)	67.9290(10)
Volume (Å3)	4403.4(14)	2095.28(13)
Z	2	2
ρcalc (g cm-3)	1.36	1.413
μ (mm-1)	0.71	0.744
F(000)	1884	924
Crystal size (mm3)	0.2 × 0.1 × 0.06	0.041 × 0.02 × 0.01
Radiation	MoKα (λ = 0.71073)	MoKα (λ = 0.71073)
2Θ range for data collection (°)	2.452 to 56.73	3.816 to 56.598
Index ranges	-15 ≤ h ≤ 15, -22 ≤ k ≤ 22, 0 ≤ l ≤ 30	-13 ≤ h ≤ 13, -15 ≤ k ≤ 15, 0 ≤ l ≤ 26
Reflections collected	27985	10384
Independent reflections	27985 [Rint = 0.0916, Rsigma = 0.1086]	10384 [Rint = 0.0732, Rsigma = 0.0782]
Data/restraints/parameters	27985/66/1169	10384/1/554
Goodness-of-fit on F2	1.043	1.027
Final R indexes [I>=2σ (I)]	R1 = 0.0765, wR2 = 0.1916	R1 = 0.0562, wR2 = 0.1053
Final R indexes [all data]	R1 = 0.1171, wR2 = 0.2142	R1 = 0.0874, wR2 = 0.1185
Largest diff. peak/hole (e Å-3)	1.02/-1.21	0.82/-0.79
Flack Parameter	N/A	N/A

Table S11 Crystal data and structure refinement for **DTE-[17-18].**

Identification code	**DTE-[17]**	**DTE-[18]**
Empirical formula	$C_{46}H_{37}N_3O_7S_2Zn$	$C_{48}H_{38}N_2O_6S_2Zn$
Formula weight	873.27	868.29
Temperature (K)	90	90
Crystal system	triclinic	hexagonal
Space group	*P*-1	$P6_422$
*a (*Å)	10.2620(11)	15.4656(4)
b (Å)	11.2119(12)	15.4656(4)
c (Å)	18.905(2)	45.0635(14)
α (°)	87.543(3)	90
β (°)	82.666(3)	90
γ (°)	66.797(3)	120
Volume (Å^3)	1982.8(4)	9334.5(5)
Z	2	6
ρ_{calc} (g cm^{-3})	1.463	0.927
μ (mm^{-1})	0.783	0.497
F(000)	904	2700
Crystal size (mm^3)	0.04 × 0.02 × 0.02	0.17 × 0.16 × 0.12
Radiation	MoKα (λ = 0.71073)	MoKα (λ = 0.71073)
2Θ range for data collection (°)	3.952 to 52.828	3.172 to 61.132
Index ranges	$-12 \le h \le 12$, $-13 \le k \le 14$, $0 \le l \le 23$	$-22 \le h \le 22$, $-22 \le k \le 22$, $-64 \le l \le 64$
Reflections collected	8125	222145
Independent reflections	8125 [R_{int} = 0.1025, R_{sigma} = 0.1196]	9557 [R_{int} = 0.0847, R_{sigma} = 0.0352]
Data/restraints/parameters	8125/1/544	9557/12/285
Goodness-of-fit on F^2	1.051	1.054
Final R indexes [I>=2σ (I)]	R_1 = 0.0693, wR_2 = 0.1156	R_1 = 0.0522, wR_2 = 0.1103
Final R indexes [all data]	R_1 = 0.1263, wR_2 = 0.1350	R_1 = 0.0704, wR_2 = 0.1172
Largest diff. peak/hole (e Å^{-3})	0.56/-0.72	0.47/-0.30
Flack Parameter	N/A	0.269(16)

Table S12 Crystal data and structure refinement for **DTE-[19].**

Identification code	**DTE-[19]**
Empirical formula	$C_{76}H_{56}N_4O_4S_4Zn_2$
Formula weight	1348.22
Temperature (K)	273.15
Crystal system	monoclinic
Space group	*I*2/*a*
a (Å)	20.2935(7)
b (Å)	16.6798(6)
c (Å)	25.1409(12)
α (°)	90
β (°)	109.8350(10)
γ (°)	90
Volume (Å^3)	8005.1(6)
Z	4
ρ_{calc} (g cm^{-3})	1.119
μ (mm^{-1})	0.748
F(000)	2784
Crystal size (mm^3)	0.1 × 0.06 × 0.02
Radiation	MoKα (λ = 0.71073)
2Θ range for data collection (°)	3.242 to 52.746
Index ranges	-25 ≤ h ≤ 25, -20 ≤ k ≤ 20, -31 ≤ l ≤ 31
Reflections collected	70213
Independent reflections	8196 [Rint = 0.0569, Rsigma = 0.0330]
Data/restraints/parameters	8196/0/408
Goodness-of-fit on F^2	1.06
Final R indexes [I>=2σ (I)]	R1 = 0.0337, wR2 = 0.0841
Final R indexes [all data]	R1 = 0.0435, wR2 = 0.0893
Largest diff. peak/hole (e Å^{-3})	0.46/-0.32
Flack Parameter	N/A
